# Diatoms on the carapace of common snapping turtles: *Luticola* spp. dominate despite spatial variation in assemblages

**DOI:** 10.1371/journal.pone.0171910

**Published:** 2017-02-13

**Authors:** Shelly C. Wu, Elizabeth A. Bergey

**Affiliations:** 1 Oklahoma Biological Survey, Norman, Oklahoma, United States of America; 2 Department of Biology, University of Oklahoma, Norman, Oklahoma, United States of America; University of Arkansas Fayetteville, UNITED STATES

## Abstract

Filamentous algae are often visible on the carapaces of freshwater turtles and these algae are dominated by a few species with varying geographic distributions. Compared to filamentous algae, little is known about the much more speciose microalgae on turtles. Our objectives were to compare the diatom flora on a single turtle species (the common snapping turtle, *Chelydra serpentina*) across part of its range to examine spatial patterns and determine whether specific diatom taxa were consistently associated with turtles (as occurs in the filamentous alga *Basicladia* spp.). Using preserved turtle specimens from museums, we systematically sampled diatoms on the carapaces of 25 snapping turtles across five states. The diverse diatom assemblages formed two groups–the southern Oklahoma group and the northern Illinois/Wisconsin/New York group, with Arkansas not differing from either group. Of the six diatom species found in all five states, four species are widespread, whereas *Luticola* cf. *goeppertiana* and *L*. cf. *mutica* are undescribed species, known only from turtles in our study. *L*. cf. *goeppertiana* comprised 83% of the diatom abundance on Oklahoma turtles and was relatively more abundant on southern turtles (Oklahoma and Arkansas) than on northern turtles (where mean abundance/state was > 10%). *L*. cf. *mutica* was the most abundant species (40%) on New York turtles. Some *Luticola* species are apparently turtle associates and results support a pattern of spatial variation in *Luticola* species, similar to that in *Basicladia*. Using museum specimens is an efficient and effective method to study the distribution of micro-epibionts.

## Introduction

Hard-surfaced, benthic substrates can be a limiting resource for attached organisms in both freshwater and marine aquatic habitats because of the abundance of organisms that can potentially settle and establish on these surfaces [1]. The consequent biofilm varies from thin coatings to upright, architecturally-rich assemblages on rocks and other submerged surfaces [2, 3]. In addition to abiotic substrates, colonized surfaces include aquatic flora and fauna, in which case, the associated biofilm organisms are referred to as epibionts.

Turtles are an excellent model to study the host-epibiont relationship in freshwaters. Turtles are large and fairly speciose with species varying in habitat use, behavior, and distribution. The most studied alga epibiont on turtles are filamentous green algae in the genus *Basicladia* (= *Arnoldiella* in [4]), species that are rare on other substrates [5, 6]. In the United States, two turtle epibionts (*B*. *chelonum* and *B*. *crassa*) often co-occur in the same regions and on the same turtle species [5, 7]. Although the complete distribution of these species is unclear, they are known from Ontario in the north [8] to Cuba in the south [9], and westward to Arizona [10]. Beyond this range, *B*. *chelonum* has been reported as a non-native species in Oregon [11] and *Basicladia* species, including *B*. *chelonum*, have been reported on turtles in South America [12, 13]. Other turtle-dwelling *Basicladia* species are found in Japan [14] and Australia [15].

In contrast to macroscopic filamentous algae, microalgae on turtle carapaces have been little studied, but recent reports indicate a combination of generalists and host specialists. Specifically, two new diatom species, *Tursiocola podocnemicola* and *Luticola deniseae* were recently described from turtles in the Amazon Basin in Brazil [16, 17] and a third species, *Mastogloia sterijovskii*, was described from a Macedonian turtle [18]. Floristic surveys of turtle-dwelling diatoms include surveys on the European pond turtle (*Emys orbicularis*) in Turkey [19, 20], with a total of 18 diatom species, a survey of two turtle species (*Pseudemys concinna* and *Trachemys scripta*) in Arkansas (USA), which lists 13 genera [21], and a list of 13 taxa on the red-headed river turtle (*Podocnemis erythrocephala*) in Brazil [17].

In contrast to ecological studies of the turtle-associated *Basicladia* (e.g.[6, 12, 22]), ecological understanding of turtle-associated diatoms is lacking. As a step in understanding the ecology of turtle-associated diatoms, our main objectives were to assess distributional patterns of diatom assemblages across the range on a single turtle species and to determine if there is an association between turtles and any particular diatom species (similar to the association of turtles with *Basicladia*). The common snapping turtle (*Chelydra serpentina*) was chosen to assess diatom assemblages across states because this species hosts (macroscopic) algae on its carapace [23–25] and has a wide distribution, covering two-thirds of the United States [26]. A secondary objective of this project was to trial the use of museum specimens to study turtle epibionts, as our study was based entirely on turtle specimens from two natural history museums.

## Materials and methods

### Turtle sampling

**Fig 1:**
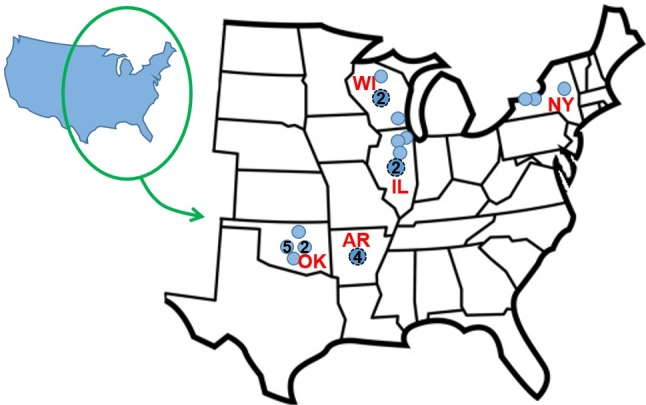
Origin of snapping turtles used to assess epibiotic algae. Numbers within dots indicate that more one turtle specimen for the location and larger circles with dashed outlines that are centered in states indicate unknown locations within states. The states map shows the majority of the turtle’s distribution in North America (e.g., NatureServe 2015). State abbreviations are: OK = Oklahoma; AR = Arkansas; IL = Illinois, WI = Wisconsin, NY = New York.

Regions chosen for this study, based on the availability of museum specimens, were Oklahoma (n = 9 turtles), Arkansas (n = 4), Illinois (n = 5), Wisconsin (n = 4), and New York (n = 3) (Fig 1). Oklahoma turtles were sampled at the Sam Noble Oklahoma Museum of Natural History in Norman, Oklahoma and turtles from other states were sampled at the Field Museum of Natural History in Chicago, Illinois (specimen data is listed S1 Table). Although we had anticipated a larger sample size based on the supplied list of museum specimens, many specimens were unsuitable because they were hatchlings/young juveniles or were road kills with smashed carapaces. Juvenile turtles have relatively few filamentous algae on their carapaces [5, 10], possibly because of their general behavior of hiding during the day, which would limit the light needed for algal growth [27].

**Fig 2:**
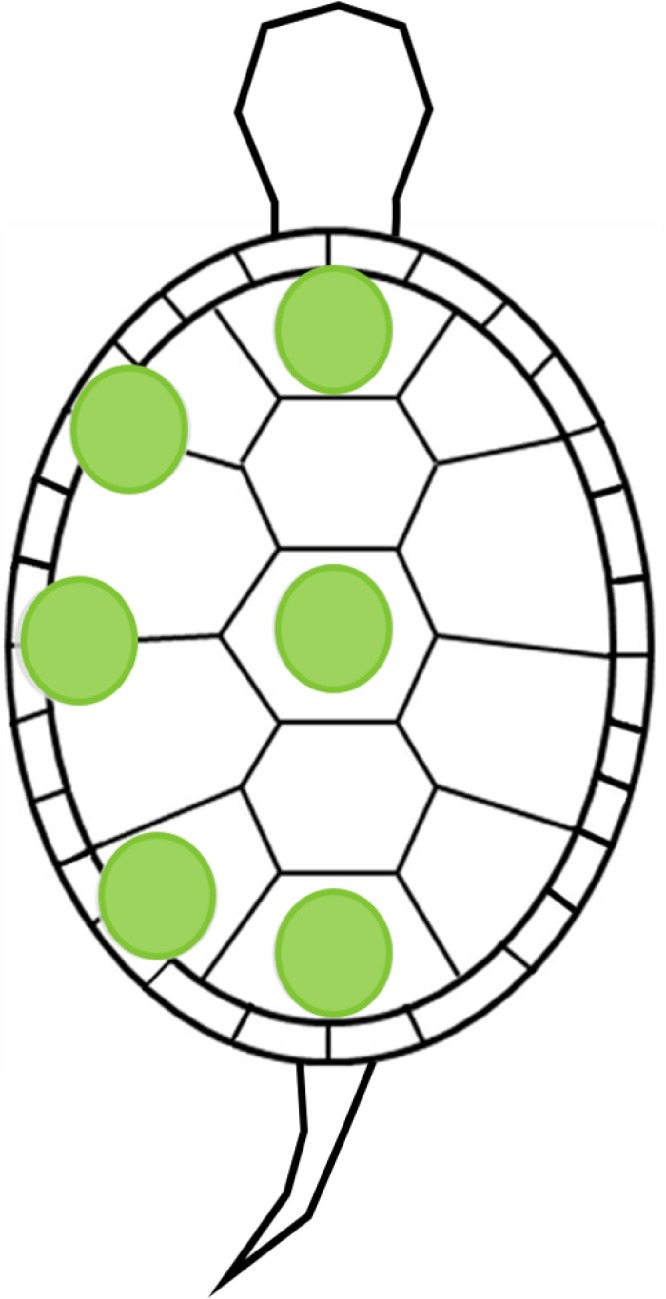
Areas sampled on the turtle carapace. Green circles represent the sampled locations: three vertebral scutes and three costal/marginal scutes.

Turtle carapaces were sampled systematically. Three vertebral scutes and a total of three costal and marginal scutes (Fig 2) were sampled by placing a plastic tube with a 2.54 cm internal diameter on each sampled scute and algae in the enclosed area were removed by brushing with a test tube brush (diameter: 1.3 cm). The total area sampled on each turtle carapace was 30.4 cm^2^. Samples were preserved in 70% ethanol. Although the use of preserved museum specimens does not require a permit, the turtle sampling protocol was approved by the University of Oklahoma Institutional Animal Care and Use Committee (tracking number R14-008).

### Diatom processing

Samples were processed to eliminate the organic material prior to diatom species identification. Samples were dried onto coverslips and coverslips were heated in a muffle furnace at 450°C for 1.5 hours. Cooled coverslips were mounted on microscope slides with Naphrax mounting medium (PhycoTech, Inc., St. Joseph, MI). Diatoms were viewed under 1000x magnification using an Olympus CX41 microscope and were identified using [28] and the Diatoms of the United States website [29]. Diatoms were counted to 200 valves by scanning transects across the coverslip. For samples with less than 200 valves, all the diatom valves in the sample were counted.

### Statistical analysis

Untransformed diatom abundance and richness data were analyzed with 1-way ANOVA (data met homogeneity and equal variance tests). Tukey tests were used to identify differences in diatom assemblages among states following a significant main test. Raw counts of diatom valves in the assemblage data set were square root transformed prior to multivariate analysis, a step that reduced the weight of less common species [30]. Non-metric Multidimensional Scaling (NMDS) was performed with Bray Curtis similarities. One way-PERMANOVA (Permutational MANOVA) tests, using type III sums of squares and 4999 permutations, were used to test for differences in diatom assemblages among states. For significant PERMANOVA results, associated pair-wise tests were used to identify significantly different states. Similarity Percentage Analysis (SIMPER) was used to identify the diatom taxa contributing the most to differences between states. PERMANOVA, NMDS, and SIMPER analyses were run with PRIMER version 6 and PERMANOVA+ packages (Primer-E Ltd, Plymouth Marine Laboratory, Plymouth, U.K.).

## Results

### Diatoms on the common snapping turtle across regions

A total of 107 diatom species were found on common snapping turtles across the five sampled states (S2 Table). Overall mean diatom richness was 12.5 +1.9 SE taxa per turtle (median = 9; range: 2–37 taxa). Only six diatom species (*Caloneis bacillum*, *Gomphonema parvulum*, *Luticola* cf. *goeppertiana*, *Luticola* cf. *mutica*, *Nitzschia amphibia*, and *Nitzschia inconspicua*) were found on snapping turtles in all five states. Of these species, *Luticola* cf. *goeppertiana* occurred on the greatest number of turtles (21 of 25 turtles). In contrast, 59 diatom species (55% of all species found on snapping turtles) occurred in only a single state.

**Fig 3:**
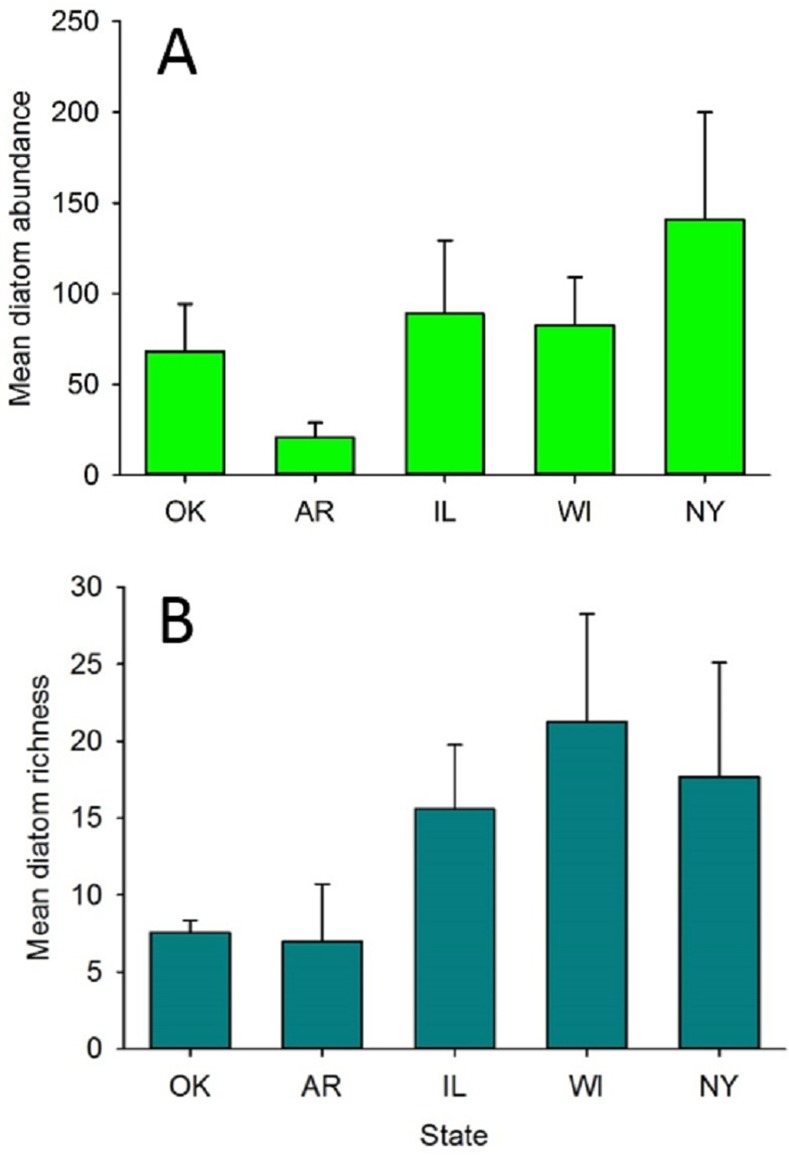
**Mean (+ SE) abundance (A) and richness (B) values for diatom assemblages on snapping turtles.** OK = Oklahoma; AR = Arkansas, IL = Illinois, WI = Wisconsin and NY = New York.

Diatom richness and abundance were variable both among and within states. As a consequence of this within state variation, neither diatom abundance nor richness was significantly different among states (ANOVA: abundance: F_4,20_ = 2.62, P = 0.066; richness: F_4,20_ = 1.18, P = 0.35, respectively). Trends were evident, however (Fig 3). Arkansas snapping turtles averaging only 20.8 (8.2 SE) diatoms per sample, whereas New York turtles averaged 141.0 (59.0 SE) diatoms. Among states, mean diatom richness per turtle was lower in Arkansas and Oklahoma (7.0 to 7.5 species) than in the other three states (15.6 to 21.3 diatom species per turtle).

*Luticola* cf. *goeppertiana* was the most common diatom on turtles in three states (OK, AR, IL), where percent abundance ranged from 27% to 83% (Table 1). In Wisconsin, *Frustulia rhomboides* (15%) was the most abundant species and in New York, *Luticola* cf. *mutica* (40%) was the most abundant. Although not the most common species, *Luticola* cf. *goeppertiana* comprised over 10% of the assemblage in both Wisconsin and New York. A few species were slight acidophiles; these included *Tabellaria flocculosa*, 9 species of *Pinnularia*, and 8 species of *Eunotia*. These 18 species were distributed across states as follows: WI (15 spp.) > IL (6 spp.) > NY (4 spp.) > OK (2 spp.) > AR (1 spp.).

**Table 1:** Percent composition of numerically dominant diatom taxa sampled on snapping turtles from the five states.

			States		
	OK	AR	IL	WI	NY
*Achnanthidium* sp. 1			+	7.9	+
*Aulacoseira granulata*	+		12.8	+	+
*Epithemia adnata*			7.4	+	
*Eunotia* sp. 1				5.8	
*Frustulia rhomboides*				15.2	+
*Luticola* cf. *goeppertiana*	83.3	34.9	26.8	10.6	13.9
*Luticola* cf. *mutica*	+	+	+	+	40.2
*Navicula cryptonella*		+	+	5.2	+
*Nitzschia frustulum*	+	18.1	+		+
*Nitzschia inconspicua*	+	+	7.4	+	+
*Pinnularia microstauron*				5.5	
Mean diatom count	68.0	20.8	88.8	82.5	141.0

Percentages are listed for the most common species that cumulatively comprise at least 50% of total diatom abundance on turtles within each state. ‘+’ indicates that the species is present, but not as a numerically dominant in turtles from the other states. Blanks indicate diatom taxa not found on the states’ turtles.

**Fig 4:**
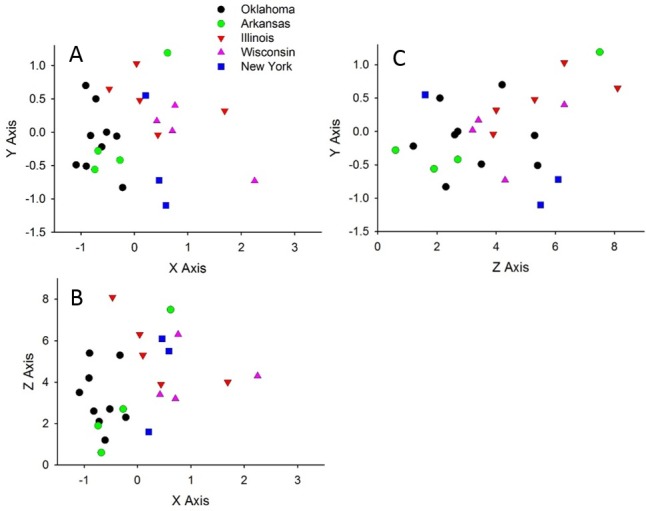
Non-metric multi-dimensional scaling plots of diatom assemblages on the shells of snapping turtles collected from five states (Oklahoma, Arkansas, Illinois, Wisconsin, and New York). The plots show the three orthogonal projections (A, B, and C) of the 3-D plot (stress = 0.17).

Diatom assemblages on the common snapping turtle were significantly different across states (PERMANOVA: pseudo-F_4,29_ = 2.20; P = 0.0002; Fig 4). Specifically, diatom assemblages on Oklahoma turtles differed from assemblages in Illinois, Wisconsin, and New York (pseudo-t: P ≤ 0.01), whereas Arkansas diatom assemblages did not differ from Oklahoma or the Illinois-Wisconsin-New York assemblages (P > 0.05). Based on SIMPER analysis, Oklahoma turtles had a higher percent abundance of *Luticola* cf. *goeppertiana* (83% of all OK diatoms) compared to Illinois (26.8%), New York (14%) and Wisconsin turtles (11%) (S3 Table; Table 1). Other taxa that contributed to differences between pairs of states include a greater abundance of *Luticola* cf. *mutica* (40%) on New York turtles compared to Oklahoma turtles (1.6%) and the high abundance of *Frustulia rhomboides* (15%) on Wisconsin turtles and this taxon’s absence on Oklahoma turtles.

## Discussion

Diatom assemblages on snapping turtles from five states showed distinct spatial patterns despite variation in collection dates and years, and differences in collection site location, if known, within states–all of which affect diatom assemblages. Oklahoma diatom assemblages differed from assemblages from the more northern states of Illinois, Wisconsin, and New York, whereas Arkansas assemblages did not differ from either of these groups. The similarity of Oklahoma-Arkansas and Illinois-Wisconsin diatoms could indicate spatial similarity in the diatom floras of adjacent states but the similarity of floras from non-contiguous states (AR versus IL-WI-NY; IL-WI versus NY), indicates other factors than proximity.

The general north-south (IL-WI-NY versus OK) difference in turtle diatom flora might be related to latitudinal differences in annual activity period by snapping turtles or biogeographical patterns in diatom distributions. Snapping turtles are occasional aerial baskers [31] that have latitudinal variation in the annual period of activity. For example, Illinois snapping turtles are active for 10 months, from February to December [32], whereas snapping turtles from Ontario, Canada are active for only 4 months, from June to October [33]. This longer activity period among southern turtles may mean greater aerial exposure of carapace algae over the active season. Diatoms are susceptible to desiccation [34] to greater aerial exposure might impact diatom assemblages. Indeed, Oklahoma and Arkansas turtles show a strong trend toward lower diatom abundance and richness than turtles in the three northern states. *Luticola* is considered as an aerophilic to subaerial genus [35–37] and the higher percent abundance of the species *Luticola* cf. *goeppertiana* in OK and AR (35% to 83%, respectively) relative to the northern states (11% to 27%) corresponds to greater aerial exposure over the annual activity period in southern versus northern states. In addition to this latitudinal pattern, diatom biogeography is affected by a combination of spatial and environmental factors [35]. Evidence for biogeographic effects in our study are that 1) 57% of the diatom taxa on snapping turtles were found in only one state and that 2) slightly acidophilic taxa, including *Tabellaria flocculosa*, *Pinnularia* species, and *Eunotia* species, occurred almost entirely in the northern states, indicating effects of regional water chemistry.

Although we found variation in the diatom assemblages among states, a survey of marine olive ridley turtles (*Lepidochelys olivacea*) from an extensive nesting area in Costa Rica found very similar diatom floras on the carapaces [38]. Olive ridley turtles nest periodically over a period of three to five months and may also coexist in feeding areas [39]. Snapping turtles in our study were collected over a wide range of years and sites (including different watersheds and different habitats); consequently, it is not surprising that the turtles in our study displayed greater variation in diatom assemblages than did the marine turtles—including a much greater species richness (our study = 107 diatom species on 25 snapping turtles; [38] = 21 diatom species on 38 olive ridley turtles).

The genus *Luticola* is a characteristic epibiont of snapping turtles. *Luticola* cf. *goeppertiana* occurred on 84% of the common snapping turtles in this study and often reached high abundances. A second species, *Luticola* cf. *mutica* was often present, but only abundant on New York snapping turtles. A third species, *Luticola deniseae*, was described from a Brazilian turtle and as in the *Luticola* in our study, *L. deniseae* dominated the epibiotic diatom flora, comprising over 80% of diatoms. *L. deniseae* was found exclusively on turtles and not on epiphytic and epilithic substrates in the same habitat [17]. One limitation to our study was that we were unable to sample other substrates in collection habitats to determine whether Luticola cf. goeppertiana occurs selectively on turtles. In contrast to the two encountered *Luticola* species in our study, the other four diatom species found in all five states (*Caloneis bacillum*, *Gomphonema parvulum*, *Nitzschia amphibia*, and *Nitzschia inconspicua*) are widely distributed species occurring on a variety of benthic substrates. More studies are needed to evaluate the degree of specificity of *Luticola*, possibly other diatom taxa on turtles, and other aquatic biota–as well as ecological studies to better understand the nature of the diatom-turtle association.

In contrast to possible specialist species in freshwaters, specialist diatom species have not been apparent on marine turtles. An SEM analysis of diatoms on single specimens of 7 marine turtle species found only 18 diatom taxa, with *Achnanthes* sp. on three turtle species, *Amphora* sp. and *Poulinea* sp. each on two turtle species [40]. Further assessment of the distribution and ecology of epizoic diatoms on both freshwater and marine turtle species may provide insights into the apparent differences in diatom specialization on turtles.

One possibility for the prevalence of *Luticola* on turtles is the ability to tolerate desiccation during aerial exposure when basking. *Luticola* is classed as a subaerial taxon [35] and like many such taxa, has occluded pores in the siliceous cell wall [41], which is an adaptation for reducing water loss [42]. The reduced openings may allow *Luticola* on turtle carapaces to tolerate aerial exposure during basking and terrestrial movement. In an experimental study that tested the tolerance of terrestrial and aquatic diatom taxa (*Luticola* were not tested) to desiccation and high temperature, all tested diatoms succumbed to desiccation but terrestrial diatoms tolerated high temperatures better than aquatic diatoms [34]. Thus, the combination of occluded pores that reduce water loss and probable tolerance to high temperatures during aerial exposure may allow *Luticola* to persist on turtle carapaces exposed to desiccation and high temperatures during basking and terrestrial movements.

*Luticola* and *Basicladia*/*Arnoldiella* are both widespread genera that include epibionts on freshwater turtles. Epibiotic species in both genera have varying geographical distributions (though tentative in *Luticola* due to the paucity of studies). Thus far, *Luticola* is known on turtles from only North and South America from this study and [17], respectively, whereas *Basicladia* has a wider distribution, occurring widely in North and South America, and in Australia [15]. Most species of *Basicladia* are turtle epiphytes (one species is found on snails and two typically occur on abiotic substrates: [9, 43], whereas most species of *Luticola* are not associated with turtles and are more typically found in soil or moss [37].

Our study was greatly facilitated by sampling museum specimens, an approach used previously for epibiotic filamentous algae on turtles [5]. Benefits of using museum collections include a combination of saving time, reducing research costs, avoiding unnecessary duplication of specimens [44], and eliminating stress to live turtles or other organisms. Limitations associated with using museum turtle specimens for studying epizoic diatoms include: 1) exact localities (and associated environmental data) are often unknown; 2) an inability to select random sampling locations or to standardize sampling variables (e.g., date of collection); and 3) possible effects of post-capture turtle processing. For example, post-capture processing would occur if museum personnel removed filamentous algae from the carapaces. Although past history of curation at the Sam Noble Museum of History is not known, algal scrapping is not a current practice and has not been documented in their database (Jessa Watters, personal communication) and algae are not routinely removed from turtles at the Field Museum (Alan Resetar, personal communication). Even if filamentous algae were removed, filamentous algae are often restricted to the sides and posterior of the carapace [5, 11] and sampling six areas of the carapace reduces any impacts on the sampled diatom assemblage. The high species richness of diatoms found in our study using museum specimens (107 species) in comparison to reported diatom richness found on live turtles (e.g. 13 to 18 taxa in [17, 20, 21]) indicates that museum specimens are a good source of epibiotic diatoms. Continuing this approach of using museum turtles and other collected taxa will allow efficient surveys of the poorly known epibionts.

## Supporting information

S1 Table List of turtle specimens sampled to assess diatom assemblages on the carapaces of snapping turtles. Museums are: OMNH = Sam Noble Oklahoma Museum of Natural History, Norman, Oklahoma; FMNH = Field Museum of Natural History, Chicago, Illinois.
(DOCX)

S2 Table Diatom data for snapping turtle samples. (XLSX)

S3 Table Summary of SIMPER pairwise comparisons of diatom assemblages on shells of snapping turtles. The table shows the five highest-contributing diatoms to differences between pairs of states with significantly different diatom assemblages (OK-IL, OK-WI, and OK-NY). SIMPER analysis was run on square-root transformed data, but data shown are untransformed mean counts (number per sample) for clarity. Diatom species occurring in all states are bolded and values in parentheses are mean counts for these diatoms that were not ranked high in the SIMPER analysis.
(DOCX)
