# Supplementary material for: Diatoms on the carapace of common snapping turtles: Luticola spp. dominate despite spatial variation in assemblages
Source: PLoS One. 2017 Feb 13;12(2):e0171910. doi: 10.1371/journal.pone.0171910 (PMC5305193; doi:10.1371/journal.pone.0171910)
Supplement: S1 Table — Museums are: OMNH = Sam Noble Oklahoma Museum of Natural History, Norman, Oklahoma; FMNH = Field Museum of Natural History, Chicago, Illinois. (DOCX) [file pone.0171910.s001.docx]

**S1 Table. List of turtle specimens sampled to assess diatom assemblages on the carapaces of snapping turtles.** Museums are: OMNH = Sam Noble Oklahoma Museum of Natural History, Norman, Oklahoma; FMNH = Field Museum of Natural History, Chicago, Illinois.

| State | Location | Collection date | Museum | Collection number |
| --- | --- | --- | --- | --- |
| Oklahoma | Cleveland County | 16 Oct 1926 | OMNH | 5577 |
| Oklahoma | Cleveland County | 16 Oct 1926 | OMNH | 5571 |
| Oklahoma | Cleveland County | 16 Oct 1926 | OMNH | 5573 |
| Oklahoma | Cleveland County | Apr 1932 | OMNH | 12877 |
| Oklahoma | Cleveland County | 12 May 1935 | OMNH | 19093 |
| Oklahoma | Murray County | 22 Aug 1951 | OMNH | 27453 |
| Oklahoma | Pawnee county | Mar 1928 | OMNH | 7991 |
| Oklahoma | Seminole County | 1931 | OMNH | 10160 |
| Oklahoma | Seminole County | May 1931 | OMNH | 10921 |
| Arkansas | unknown | 1 May 1926 | FMNH | 8812 |
| Arkansas | unknown | 1 Jun 1926 | FMNH | 8939 |
| Arkansas | unknown | 1 Jun 1926 | FMNH | 8941 |
| Arkansas | unknown | 1 Jun 1926 | FMNH | 8942 |
| Illinois | Cook County | 1 Aug 1941 | FMNH | 164575 |
| Illinois | Du Page County | 24 Jun 1934 | FMNH | 22738 |
| Illinois | Grundy County | 1 Jun 1941 | FMNH | 37198 |
| Illinois | unknown | Sep 1922 | FMNH | 3291 |
| Illinois | unknown | 4 Jun 1925 | FMNH | 8108 |
| Wisconsin | Oneida County | 15 Aug 1936 | FMNH | 24224 |
| Wisconsin | Racine County | 1 Jul 1946 | FMNH | 164577 |
| Wisconsin | unknown | 21 Jul 1929 | FMNH | 14717 |
| Wisconsin | unknown | 14 Aug 1927 | FMNH | 13057 |
| New York | Monroe County | 11 Jul 1931 | FMNH | 92010 |
| New York | Saratoga County | 8 Jun 1932 | FMNH | 92007 |
| New York | Wayne County | 14 Jul 1929 | FMNH | 92006 |
