# Supplementary material for: Diatoms on the carapace of common snapping turtles: Luticola spp. dominate despite spatial variation in assemblages
Source: PLoS One. 2017 Feb 13;12(2):e0171910. doi: 10.1371/journal.pone.0171910 (PMC5305193; doi:10.1371/journal.pone.0171910)
Supplement: S3 Table — The table shows the five highest-contributing diatoms to differences between pairs of states with significantly different diatom assemblages (OK-IL, OK-WI, and OK-NY). SIMPER analysis was run on square-root transformed data, but data shown are untransformed mean counts (number per sample) for clarity. Diatom species occurring in all states are bolded and values in parentheses are mean counts for these diatoms that were not ranked high in the SIMPER analysis. (DOCX) [file pone.0171910.s003.docx]

**S3 Table. Summary of SIMPER pairwise comparisons of diatom assemblages on shells of snapping turtles.** The table shows the five highest-contributing diatoms to differences between pairs of states with significantly different diatom assemblages (OK-IL, OK-WI, and OK-NY). SIMPER analysis was run on square-root transformed data, but data shown are untransformed mean counts (number per sample) for clarity. Diatom species occurring in all states are bolded and values in parentheses are mean counts for these diatoms that were not ranked high in the SIMPER analysis.

|  | States | | | | |
| --- | --- | --- | --- | --- | --- |
|  | OK | AR | IL | WI | NY |
| *Aulacoseira granulata* | 0.1 |  | 11.4 |  |  |
| *Cocconeis placentula* | 0.0 |  |  |  | 3.3 |
| *Eunotia incisa* | 0.2 |  |  | 2.0 |  |
| *Fragilaria capucina* | 0.0 |  | 4.2 |  |  |
| *Frustulia rhomboides* | 0.0 |  |  | 12.5 |  |
| ***Luticola* cf. *goeppertiana*** | 56.7 | (7.3) | 23.8 | 8.8 | 19.7 |
| ***Luticola mutica*** | 0.1 | (1.8) | (0.2) | (1.3) | 56.7 |
| ***Nitzschia amphibia*** | 0.0 | (0.3) | (2.0) | (1.0) | 5.7 |
| *Nitzschia frustulum* | 0.3 |  | 2.6 |  |  |
| ***Nitzschia inconspicua*** | 0.1 | (0.3) | 6.6 | 4.0 | (9.3) |
| *Pinnularia microstauron* | 0.0 |  |  | 4.5 |  |
| *Planothidium lanceolatum* | 0.2 |  |  |  | 7.7 |
| ***Caloneis bacillum*** | (0.9) | (0.3) | (3.8) | (0.8) | (0.3) |
| ***Gomphonema parvulum*** | (0.2) | (0.3) | (1.2) | (2.8) | (0.3) |
| Mean diatom count | 68.0 | 20.8 | 88.8 | 82.5 | 141.0 |
